# In the lab and in the wild: How distraction and mind wandering affect attention and memory

**DOI:** 10.1186/s41235-018-0137-0

**Published:** 2018-11-21

**Authors:** Trish L. Varao-Sousa, Daniel Smilek, Alan Kingstone

**Affiliations:** 1 Department of Psychology, University of British Columbia, Vancouver, BC Canada; 2 Department of Psychology, University of Waterloo, Waterloo, ON Canada

**Keywords:** Mind wandering, Distraction, Natural environments, Everyday attention, Audiobooks

## Abstract

The present study examined the impact that the environment has on the ability to remain attentive and retain information. Participants listened to an audiobook in either a controlled lab setting or in an uncontrolled natural setting. While listening to the audiobook, participants were randomly prompted to report their current attentional status (focused, mind wandering, or distracted). Participants performed a memory test on audiobook content at the end. Inattention (mind wandering and distraction) did not differ between the two settings. However, there was a setting by attentional state interaction: distraction rates were higher than mind wandering rates outside the lab, while inattention rates did not differ inside the lab. Memory test performance was poorer outside the lab, suggesting that increased distraction may compromise memory more than mind wandering. Collectively, the data suggest that mind wandering and distraction are distinct types of attentional failures and that past controlled lab investigations may have overestimated the role of mind wandering and underestimated the role of distraction in everyday cognition.

## Significance

Everyday behavior requires individuals to manage their attention in competition with both internal (wandering thoughts) and external (environmental) events. In everyday life, a wide array of unpredictable external events may occur. However, in laboratory studies, external events are experimentally constrained. Thus, when studying internally and externally related failures in attention, the environment may influence the ability to manage attention. This study examines the influence of mind wandering and distraction on an activity that can be measured in or out of the lab: listening to an audiobook. We hypothesized that attention would be allocated differently based on whether a participant was listening to an audiobook inside or outside the lab and that this attentional variance would influence performance on a memory test. We found that while overall inattention did not differ when inside versus outside the lab, the distribution of inattention was impacted by setting. Specifically, mind wandering and distraction were the same inside the lab, but distraction was greater than mind wandering outside the lab. Additionally, overall memory performance was worse outside the lab, suggesting that distraction may be more detrimental to memory than mind wandering. In sum, by venturing beyond the typical confines of the lab, we find that past laboratory studies may have overestimated the role of mind wandering on everyday human performance and underestimated the role of distraction.

## Background

When reading a novel on the bus, one’s attention may shift away from reading to thinking about things such as what to prepare for dinner or noticing the ring tone of a nearby passenger’s phone. Such shifts of attention can be either inward, toward one’s own thoughts (i.e. mind wandering) or outward, toward an external event (i.e. a distraction). In our daily lives, the environments in which we process information can vary greatly (e.g. the number of people, peripheral sights and sounds, and secondary tasks) and these variations may influence lapses of attention in different ways. The present paper is concerned with how different task settings (i.e. research laboratory versus everyday life) influence mind wandering, distraction rates, and memory performance.

When our attention is not focused on a primary task, our ability to perform that task suffers. Lapses of attention have been associated with both self-reported cognitive errors and objective sustained attention performance failures (Cheyne, Carriere, & Smilek, 2006). Performance impairments have also been observed for specific types of attention lapses, i.e. mind wandering and distraction. In a variety of tasks (e.g. sustained attention, visual search, reading, or watching lectures), higher rates of mind wandering are often associated with greater detriments to performance (e.g. slower reaction times, greater errors, poorer memory retention; Dixon & Li, 2013; Forster & Lavie, 2009; McVay & Kane, 2009; Smallwood, McSpadden, & Schooler, 2008; Varao Sousa, Carriere, & Smilek, 2013). Similarly, distractions have been reported to negatively impact performance (e.g. poorer memory retention, slower reaction times; Banbury & Berry, 1998; Forster & Lavie, 2007; Reinten, Braat-Eggen, Hornikx, Kort, & Kohlrausch, 2017).

Researchers have also investigated the impact of, and relation between, mind wandering and distraction within a single task. Stawarczyk, Majerus, Maj, Linden, and D’Argembeau (2011) report that increased rates of mind wandering and distraction during the Sustained Attention to Response Task were related to increased response variability and errors. Additional research by Unsworth and colleagues (Robison & Unsworth, 2015; Unsworth, Brewer, & Spillers, 2012; Unsworth & McMillan, 2014; Unsworth, McMillan, Brewer, & Spillers, 2012) suggests that mind wandering and distraction are separate constructs, with distractions interfering with performance “over and above that accounted for by general lapses of attention” (Unsworth & McMillan, 2014, p. 23). Collectively, this research suggests that both distractions and mind wandering influence performance (but see Olivers & Nieuwenhuis, 2005).

What remains unclear, however, is whether the effects found in laboratory settings fully capture the role of mind wandering and distractions in everyday life. While some researchers have tried to address concerns about unnatural laboratory settings by using less controlled, more naturalistic distraction stimuli (i.e. a background television program or restaurant audio track; Pool, Koolstra, & Voort, 2003; Robison & Unsworth, 2015), these distractions are still limited to scheduled experimental events from a limited stimulus set. In day-to-day life, distractions are often more unpredictable than those experienced in a laboratory experiment.

Notably, some studies have revealed important differences in patterns of inattentiveness across laboratory and everyday settings (Kane et al., 2017; Wammes & Smilek, 2017). For instance, Kane et al. (2017) measured mind wandering rates in the laboratory while participants completed a variety of executive-control paradigms (e.g. the sustained attention to response task, the attention flanker task). These results were compared to results obtained in everyday life using experience sampling methods whereby participants responded to thought-probes on a handheld device. Two key points of divergence between mind wandering in the laboratory and in life were observed. First, mind wandering rates during laboratory tasks did not correlate with mind wandering rates collected during everyday tasks, leading the authors to conclude that “any relation between laboratory and overall daily-life mind-wandering propensities is not robust” (p. 1278). Second, while measures of executive control were related to mind wandering rates in the laboratory, they were not directly related to mind wandering rates outside of the laboratory.

The divergence between life and lab is also supported by a recent study by Unsworth and McMillan (2017) in which participants completed in-lab cognitive ability tests and then, for one week, reported mind wandering and distractions that took place while studying or while in class. In contrast to laboratory studies showing that mind wandering and distraction negatively influence performance (Banbury & Berry, 1998; Dixon & Li, 2013; Forster & Lavie, 2007; Forster & Lavie, 2009; McVay & Kane, 2009; Reinten et al., 2017; Smallwood et al., 2008; Varao Sousa et al., 2013), their results suggest that everyday reports of mind wandering and distraction were not correlated with in-lab cognitive ability measures or with academic performance. However, it is worth noting that there were exceptions in Unsworth and McMillan (2017), such that some categories of mind wandering and distraction did correlate with in-lab measures (e.g. “Mind wandering due to disinterest” correlated with most in-lab cognitive tasks, while “Distraction due to hunger” did not) (see also Kane et al., 2017; Kane et al., 2007; Unsworth, Brewer, & Spillers, 2012). These results suggest that the negative consequence of attentional failures is not absolute and varies with task setting. This dovetails with the idea that the true diversity and impact of inattention in everyday environments may not have been captured by previous studies of mind wandering and distraction in the lab.

Studies of mind wandering during university lectures also show different patterns of mind wandering between lab and life settings. When students are asked to watch video recordings of lectures in the laboratory, mind wandering rates typically increase as the lecture progresses (Farley, Risko, & Kingstone, 2013; Risko, Anderson, Sarwal, Engelhardt, & Kingstone, 2012; Wammes & Smilek, 2017). In contrast, when attending a live lecture, mind wandering rates of undergraduates remain stable across the lecture (Wammes, Boucher, Seli, Cheyne, & Smilek, 2016; Wammes & Smilek, 2017).

Given recent results indicating a divergence in mind wandering rates across laboratory and real-life situations, determining why such a divergence occurs becomes an issue. One plausible reason for the divergence, which we focus on here, concerns the availability of distractions in the laboratory and in everyday life. In the laboratory, testing situations are typically controlled, with very few distractions available to co-opt attentional resources. In fact, laboratory settings are specifically designed to reduce and control distraction. In such cases, mind wandering is often the primary way (if not the only way) that inattention can be manifested. In contrast, within the natural world, opportunities for distraction seem frequent and diverse (e.g. the presence of other people, peripheral sights and sounds, media devices). In such a complex setting, inattention may be expressed in ways other than mind wandering. If the rate and impact of inattention differ between the lab and the outside world, previous lab research may be underestimating the impact that inattention has in uncontrolled environments.

To determine whether rates of inattention differ when inside and outside of the lab, we manipulated whether participants listened to an audiobook while inside the lab (controlled setting) or outside the lab (uncontrolled setting). We allowed participants to move freely outside the lab to reduce the constraints of a lab testing room and amplify the “noise” due to the perceptual richness or affordances found in everyday environments. We chose an audiobook task for two reasons: (1) mind wandering occurs frequently and reliably in this setting (Kopp & D’Mello, 2015; Varao Sousa et al., 2013); and (2) an audiobook allows one to move freely while still completing the primary task of listening. While listening, participants were prompted by an audio tone to report whether they were on task, mind wandering, or distracted. With this design we could compare rates of mind wandering and distraction in an uncontrolled everyday environment with those in a controlled lab environment. The decision to allow participants to move freely while outside the lab was in keeping with the fundamental methodology of cognitive ethology (Kingstone, Smilek, Birmingham, Cameron, & Bischof, 2005; Kingstone, Smilek, & Eastwood, 2008; Smilek, Birmingham, Cameron, Bischof, & Kingstone, 2006). Our goal was to first discover what people naturally do in an everyday environment and how that impacts cognition. Thus, participants were not instructed on how they should behave outside the lab, anticipating that choices made would reflect normative behavioral responses.

We predicted that rates of inattention would not be equivalent between life and lab environments. Specifically, we predicted participants outside the lab would report more instances of distraction, relative to mind wandering, than those inside the lab, as the former affords exposure to a greater array of dynamic stimuli. Since mind wandering is an internally driven process, we had no reason to predict that different external environments would influence the rates of mind wandering. Since Unsworth and McMillan (2014) found that distractions impaired memory test performance, and that we predicted greater distraction outside the lab, we further predicted that memory test performance should be worse when the audiobook was listened to outside the lab than inside. We also conducted an exploratory analysis to see if self-reported ratings of interest, motivation, or boredom (factors commonly correlated with mind wandering) were influenced by the task setting or were related to these attention measures.

## Method

Below we report how we determined our sample size, all data exclusions, all manipulations, and all measures in the study (Simmons, Nelson, & Simonsohn, 2012).

### Participants

Sample size (*n* = 34/group) was determined by conducting a power analysis using G*Power software (Faul, Erdfelder, Lang, & Buchner, 2007). This analysis was based on obtaining 90% power to detect the effect size (*d* = 0.78) found in Robison and Unsworth (2015), who looked at differences in mind wandering and distraction in a laboratory setting. Seventy-three students, 12 men and 61 women, aged 18–42 years (*M* = 21.32, *SD* = 3.57) from the University of British Columbia participated in the study. Participants were compensated with course credit. Eight participants were dropped from the analysis due missing responses (i.e. did not respond to all the probes), leaving 34 in the Inside (controlled) setting and 31 in the Outside (uncontrolled) setting.

### Materials and measures

#### Audiobook

A 14-min excerpt from *Weapons of Math Destruction* (O’Neil, 2016) narrated by the author was used. The audiobook was presented on a fourth-generation iPod Shuffle, with a generic pair of headphones.

#### Probes

Ten audio tones were dispersed roughly 1–2 min apart throughout the audio track. A sample of the audio probe was presented to participants during the instructions. Participants were provided a numbered response sheet and responded to the audio probes by circling one of the following options after each tone: “I am focused on the audiobook,” “I am distracted by sights/sounds,” and “I am mind wandering.”

#### Memory test

A memory test on the contents of the audiobook was administered via an online Qualtrics survey. The memory test consisted of nine multiple choice questions and one short answer question.

#### Other measures

Ratings of motivation to attend the audiobook and interest in the material were collected using a five-point Likert scale. Boredom, as a state of disengagement, was measured via the seven-point Likert “Short Boredom Proneness Scale” (Struk, Carriere, Cheyne, & Danckert, 2017). Participants were also asked about their experience during the task (what they did while listening, whether the task reflected real-world audiobook listening, and how often they listened to audiobooks). Participants assigned to the Outside condition were additionally asked where they went, what proportion of time they spent outside, and if they interacted with anyone (see Appendix 1, 2 and 3 for all questions and responses). All questions were administered via Qualtrics.

### Procedure

After providing informed consent, participants were randomly assigned to either the Inside or Outside condition. Participants assigned to the Inside condition remained inside a laboratory testing room for the duration of the audiobook track, while participants assigned to Outside condition were allowed to move freely anywhere outside the laboratory. The iPod “lock” feature was enabled so that participants could not pause, rewind, or skip through the audio track. Participants in the Outside condition were advised to return to the lab once they had heard and responded to the tenth and final mind wandering probe, while continuing to listen to the audiobook. Participants in the Outside condition who arrived with time remaining on the audiobook track were taken to the testing room to listen to the remainder of the track. Once the audiobook track had finished, the Qualtrics survey was administered.

## Results

All statistical analyses are reported with two-tailed *p* values. In cases where the samples were not homogeneous, as indicated by Levene’s test of homogeneity, an adjusted degrees of freedom value is reported. Overall inattention was calculated by summing mind wandering and distraction rates. Table 1 shows descriptive statistics for all variables measured.

**Table 1:** Descriptive statistics split by condition

Condition	Measure	Mean	SE	SD	Kurtosis	Skew
Inside (*N* = 34)	Distraction rate	0.18	0.03	0.17	0.93	− 0.13
	Mind wandering rate	0.20	0.02	0.13	0.19	− 0.28
	Overall inattention	0.38	0.03	0.19	− 0.14	− 0.71
	Memory	0.62	0.03	0.16	− 0.18	0.35
	Interest	2.35	0.13	0.78	0.11	− 0.21
	Motivation	3.03	0.19	1.09	− 0.51	− 0.66
	Boredom	3.26	0.17	0.96	0.30	− 0.69
Outside (*N* = 31)	Distraction rate	0.24	0.02	0.12	− 0.45	− 0.38
	Mind wandering rate	0.14	0.02	0.10	0.32	− 0.80
	Overall inattention	0.37	0.03	0.18	− 0.28	− 0.08
	Memory	0.50	0.03	0.19	− 0.31	− 0.62
	Interest	2.26	0.15	0.82	0.27	− 0.21
	Motivation	3.26	0.16	0.89	− 1.15	0.78
	Boredom	3.01	0.16	0.90	0.41	− 0.64

### Attention ratings

**Fig. 1:**
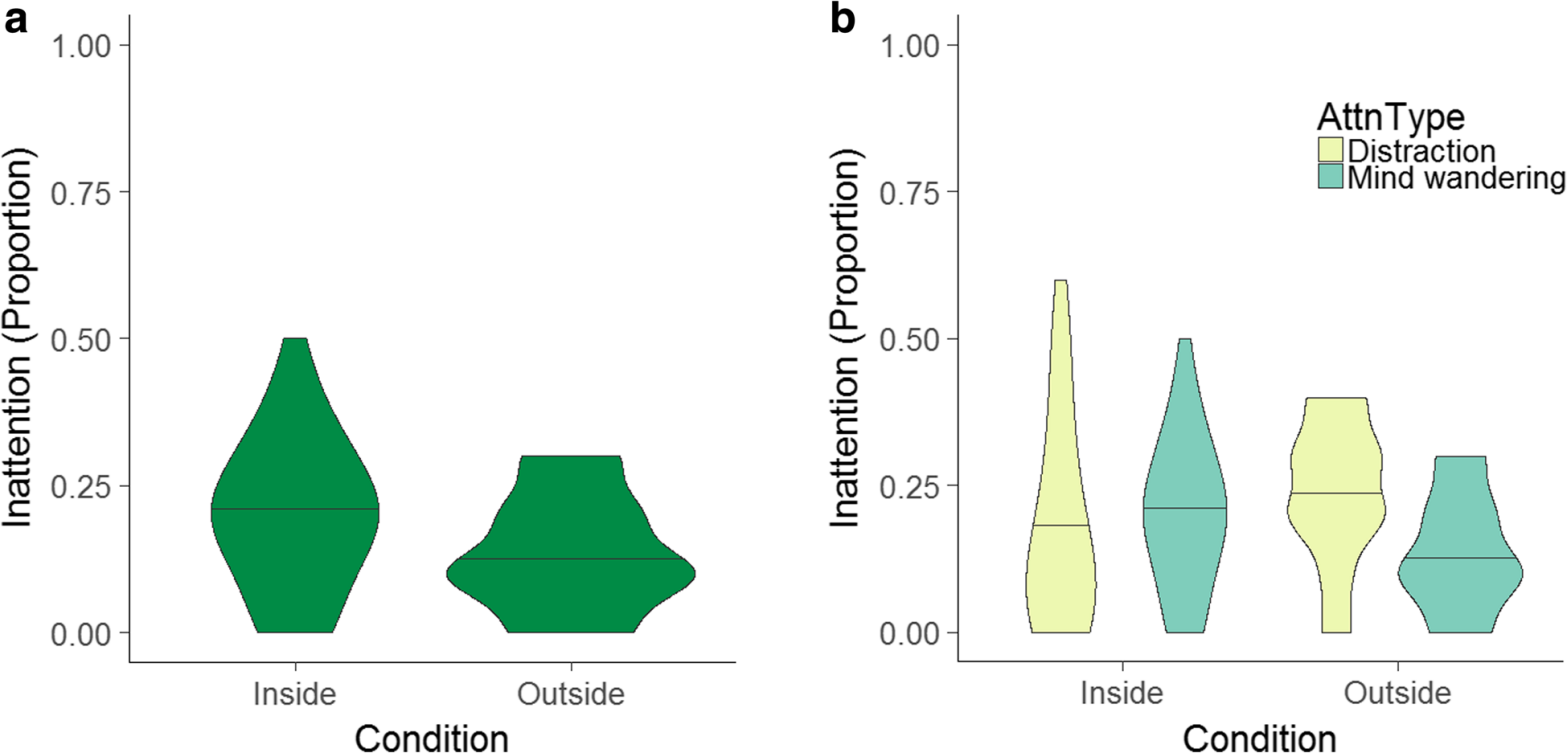
**a** Violin plots indicating density of distributions of inattention across conditions. **b** Violin plots indicating density of distributions of inattention type across conditions

A 2 × 2 between-within ANOVA assessed whether the condition (Inside or Outside) influenced mind wandering or distraction ratings.^1^ As indicated in Fig. 1a, the between-subjects test indicated that overall inattention did not differ when Inside versus Outside, *F*(1, 63) = 0.003, *p* = 0.96. There was also no within-subjects main effect of report type, *F*(1, 63) = 2.30, *p* = 0.14. However, an interaction revealed that differences between specific inattention rates (i.e. distraction or mind wandering) varied with condition, *F*(1, 63) = 6.75, *p* = 0.012. Figure 1b illustrates the follow-up, paired t-tests which indicate that mind wandering and distraction rates did not differ for those Inside, *t*(33) = 0.57, *p* = 0.57, *d* = 0.099; however, participants Outside reported significantly more inattention due to distraction than mind wandering, *t*(30) = 4.41, *p* < 0.001, *d* = 0.81.

### Memory test performance

There was a significant difference in memory based on condition, such that those Inside performed significantly better than those Outside, *t*(59.33) = 2.78, *p* = 0.007, *d* = 0.69.

### Interest, Motivation, and Boredom scales

None of the Interest, Motivation, or Boredom ratings differed between the Inside and Outside conditions; all *p* values > 0.28 (see Table 1 for descriptive statistics).

### Correlations

Table 2 displays correlations among the variable of interest. Relations between mind wandering and distraction were in the same direction for all variables; however, not all reached significance. Distraction rates while Inside were negatively correlated with both interest ratings and memory performance, such that increased reports of distraction were associated with lower memory test performance as well as lower ratings of interest. Mind wandering rates while Inside were negatively correlated with motivation ratings, suggesting that lower motivation is associated with more frequent mind wandering reports. For participants in the Outside condition, none of the relationships reached statistical significance. The relatively small samples in these correlations demand that they be viewed merely as exploratory and suggestive.

**Table 2:** Correlation table split by condition

Condition		Memory	Interest	Motivation	Boredom
Inside (*N* = 34)	Distraction (%)	− 0.44**	− 0.41*	− 0.30	0.22
	Mind wandering (%)	− 0.03	− 0.21	− 0.39*	0.25
Outside (*N* = 31)	Distraction (%)	− 0.19	− 0.34	− 0.25	0.21
	Mind wandering (%)	− 0.06	− 0.08	− 0.22	0.07

* *p* < 0.05

** *p* < 0.01

### Audiobook listening frequency

When asked how often they listen to audiobooks, 24 participants stated “Never,” 28 reported very seldom or rarely listening, seven stated a couple times per year, and six said once per month or of a greater frequency. For those who reported listening to audiobooks more than “Never,” we asked if the task reflected how they would normally listen to an audiobook. Sixty-one percent of those in the Inside condition (*n* = 23) and 56% of those in the Outside condition (*n* = 18) reported that it did. For those who felt that the task did not reflect their normal audiobook listening experience, the most common reason given was that they normally did something additional (chores, driving, on a bus, etc.) while listening.

### Other variables

Both groups were asked what they did while listening to the audiobook; these responses can be found in Appendix 2. A majority of those in the Outside condition indicated walking around inside the building or sitting down, while those in the Inside condition mainly reported looking around the room. Outside participants also reported how long they spent outside; however, responses were not clearly identified as outside the laboratory or outside the building and so this question could not be assessed further. A majority (*n* = 25) of those in the Outside condition stated that they did not interact with anyone, a few (*n* = 5) reported brief interactions, and one individual reported having a 5-min conversation.

## Discussion

The present study examined how the rates of two forms of inattention – mind wandering and distraction – vary with environment, i.e. an uncontrolled natural setting and a controlled laboratory setting. Our results suggest that overall rates of inattention are similar when one is outside in a dynamic real-world setting or inside a controlled laboratory, but there are differences in whether inattention manifests as mind wandering or as distraction. While there were no differences between mind wandering and distraction when inside the lab (convergent with Robison & Unsworth, 2015), participants outside reported more distraction than mind wandering (convergent with daily diary studies: Unsworth, Brewer, & Spillers, 2012; Unsworth & McMillan, 2017).

A secondary goal was to examine whether mind wandering and distraction rates differentially impact memory performance. As predicted, distraction rates were higher and memory test performance was worse for those outside than inside the laboratory. Exploratory correlational analyses hint that distraction, but not mind wandering rates, were significantly related to memory test performance. This preliminary observation suggests that performance may depend more on level of external distraction than on level of mind wandering, although follow-up research with larger correlation samples is required to confirm, or disconfirm, this possibility.

By applying the fundamental methodology of cognitive ethology (Kingstone et al., 2005, 2008; Smilek et al., 2006) we explored how allowing people to freely direct their attention in an everyday environment impacts various forms of inattention. With these data now in hand, future research could benefit from a more controlled design whereby participants are provided with specific instructions regarding their out-of-lab behavior. For instance, although participants’ chose to walk within the building or sit down in one location suggests these may be the normative behavioral responses, the impacts of these behaviors on attention could be explored experimentally. For example, one could systematically combine walking and sitting with visually quiet and dynamic settings to tease apart the relative contributions of these variables on different forms of inattention and their impacts on memory.

Future work would also benefit from moving beyond mind wandering and external distraction in the context of listening to audio audiobooks and examine inattention in the context of other everyday tasks, such as completing homework, driving, or preparing dinner, where individuals frequently engage in multitasking. Although prior research has examined inattention in the context of complex tasks such as driving and real-world learning settings (e.g., Cowley, 2013; Dingus et al., 2016; Unsworth & McMillan, 2017; Yanko & Spalek, 2013), these studies have not examined the distinct roles of mind wandering and distraction. The unique nature of audiobooks (lack of reliance on motor or ocular focus) invites multitasking and it is possible that audiobook users may be more susceptible to distractions than alternative tasks that requires more focused attention (e.g. silent reading). With greater task freedom, individuals may be able to anticipate or choose how to navigate their surroundings and thus optimize their task experience, thereby modulating the consequences of inattention.

One limitation to the present study is that it is unclear to what extent the behavior observed outside the laboratory truly represents real-life behavior. Many audiobooks users reported that the task did not completely reflect their “real-life” audiobook listening, noting that a secondary task would normally be completed while listening (e.g. driving, chores, etc.). This suggests that our uncontrolled, out-of-lab setting may still not be entirely capturing real-life behavior, as it lacked the natural conditions under which participants would normally complete the audiobook listening task. It would be prudent to conduct follow-up studies that include a series of settings that reflect common audiobook listening contexts (see Appendix 3).

In summary, motivated by recent research suggesting that results found in the laboratory may not generalize to everyday behavior (Kane et al., 2017; Unsworth & Mcmillan, 2017; Wammes & Smilek, 2017), the present study examined how mind wandering and external distractions differ between a dynamic, out-of-lab setting and a controlled, in-lab setting. In line with Unsworth and colleagues (Robison & Unsworth, 2015; Unsworth & McMillan, 2014; Unsworth, McMillan, Brewer, & Spillers, 2012), the current study substantiates the finding that mind wandering and external distractions are two unique types of attentional failures. Furthermore, the allocation of inattention depends on the environment: distraction was experienced more often than mind wandering outside the laboratory, while the rates of distraction and mind wandering were equivalent inside the laboratory. Additionally, memory performance was worse when outside than inside the lab. In sum, the present study suggests that research taking place within a laboratory environment may not reflect the true impact of dynamic real-world environments on inattention and retention.

## Appendix 1

### End-of-study questionnaire

All participants:

1. How motivated were you to remain attentive to the audiobook? (1–5 Likert Scale)
2. How interesting did you find the material presented in the audiobook (1–5 Likert Scale)
3. What did you do while listening to the audiobook? (Open-ended)
4. Did the study reflect how you would listen to audiobooks in the real world? (Yes/No)
5. If no, how did this setting differ from how you would normally listen to an audiobook? (Open-ended)
6. How often do you listen to audiobooks? (Open-ended)

For those in the Outside condition only:

7. Once you left the lab, where did you go and what did you do? (Open-ended)
8. What proportion of time did you spend outside? (Open-ended)
9. Did you interact with anyone and for how long? (Open-ended)

## Appendix 2

**Table 3:** Free-form responses to end-of-study questionnaire Question 1 (“What did you do while listening to the audiobook?”)

Inside condition:	
• Played around with the pen, tried to stare at the phrase “I am focused on the audio” so I can get myself to focus better, sometimes mind wandering regarding my other assignments	
• Looked around the room, fidgeted	
• Look at the ground	
• I sat with my eyes closed in order to focus effectively	
• Shake my leg, play with the pen, close my eyes	
• Looked around the room	
• Either trying to focus on retaining information or thinking about other unrelated topics	
• Expecting the tone, staring at the number on the green door	
• Tried to listen but at times I ended up playing with the zippers on my jacket	
• Stared at the clipboard	
• Fiddling with the iPod, checking phone, observing the room	
• Staring at the wall	
• Think	
• Played with my fingers and the pencil	
• I was thinking about my runny nose and a random itchy spot on my leg and looking at the green door	
• Checked my phone and thought about other things on my mind	
• Didn’t move and tried not to look around and focus on the audiobook	
• Watching the paper sheet	
• Nothing else just listen	
• When I was wasn’t listening to the audiobook I was looking around the room and would occasionally mind wander about the rest of my day	
• I checked my phone once and my mind wandered and I got distracted from sounds in the environment	
• Close my eyes and focus on the audiobook	
• Looked around the room	
• Listening but mind wandering sometimes as I thought about when the interruption sound would occur	
• I was handed a sheet of paper where I was asked to circle from a choice of three options every time I heard a sound	
• Looked at the objects around the room	
• Bored	
• Close my eyes, play with the little pencil, look at the iPod	
• Looked around the room, played with the pencil	
• I looked at the sheet in front of me or looked at the wall	
• Looked around the room or at the pencil	
• Bit my nails	
• Look at the clipboard	
Outside condition:	
• Sitting and relaxing	
• Sat in an outdoor staircase	
• I walked around the Kenny building, went and stood outside for a minute or two, then came back inside and stood looking out a window	
• Walking around	
• Walk around the building up and down the stairs	
• I walked around outside	
• Sat on the first floor of Kenny	
• Watched a tv screen in the Kenny building, people watching	
• Walked around the inside of Kenny building	
• Sat in the lobby	
• Walking	
• I was sitting down	
• Took the elevator, sat on a couch	
• Standing in the Kenny building	
• Sit down	
• Walked around the Kenny building	
• Walked around campus	
• I walked for a bit, but I was mostly sitting down	
• Walked around	
• I sat on a coach in the first floor of Kenny	
• Walking	
• Walked around Kenny	
• Sat at the second floor of Kenny, looked at my phone	
• Sat at a bench in Kenny	
• Walk around and sit down	
• Walked around and looked outside the window	
• Kenny, second floor	
• Sitting on a couch	
• I walked around and then ate lunch	
• Looking around	
• Stand on the hallway	

## Appendix 3

Free-form responses to end-of-study questionnaire Question 3 (“How did this setting differ from how you would normally listen to an audiobook?”)

Inside condition:

- I don’t sit as comfortably and I don’t write down notes. I prefer to have texts that I can follow while listening and I type my notes
- Would probably be multitasking with some kind of chore
- I would listen to an audiobook with a drink and in a more comfortable setting
- The context is boring
- I am very tired from the day, therefore less interested and the topic was not interesting to me
- I would possibly be on a bus or in a car where it would be inconvenient to carry around a book
- I usually do some chores while listening to audiobooks
- I do not listen often to audiobooks
- I usually listen to audiobooks when I am sitting on the car and have nothing else to do
- I am always doing something else apart from listening to an audiobook
- I would pick another topic for an audio book and I like to do other things while listening
- I would normally listen to an audiobook while I drive or work

Outside condition:

- I would normally listen to them on a bus ride or in a quiet setting
- I typically listen to audiobooks (well, podcasts really) when I’m cooking (e.g. chopping vegetables) or eating a meal at home
- The noise will be more longer and consist
- I would probably be sitting in my room listening to the audiobook while doing a simple or familiar task, such as drawing
- I would listen somewhere quiet and comfortable where there weren’t people around me talking and walking
- I don’t listen to audiobooks but would choose to do so at home
- I was too focusing on audiobooks, but somehow I was distracted by noise and mind wandering
- I usually listen to audio books in my bed before I sleep
- Typically I only listen to audiobooks when driving on relatively straight roadways, but having so many directions to walk in Kenny, or potentially outside, made focusing on the presented material more difficult
- I don’t know, I do not listen to audiobooks
- I would probably listen to an audiobook while drawing or commuting; content
- I do not listen to audiobooks
- I usually am in my room on my bed
- I usually lay in bed just listening to the audiobook

## Footnotes

1 We recognize that the reports are partially ipsative in nature given the three forced-choice alternative (mind wandering, distracted, on-task). We nonetheless made an a priori decision to focus our analysis on differences between types of inattention and thus follow the protocol of similar analyses (Robison & Unsworth, 2015; Unsworth & McMillan, 2014)
